# Virtual Screening for Novel SarA Inhibitors to Prevent Biofilm Formation of *Staphylococcus aureus* in Prosthetic Joint Infections

**DOI:** 10.3389/fmicb.2020.587175

**Published:** 2020-11-05

**Authors:** Jinlong Yu, Feng Jiang, Feiyang Zhang, Yunqi Pan, Jianqiang Wang, Pei Han, Jin Tang, Hao Shen

**Affiliations:** ^1^Department of Orthopedics, Shanghai Sixth People’s Hospital, Shanghai Jiao Tong University, Shanghai, China; ^2^Department of Clinical Laboratory, Shanghai Sixth People’s Hospital, Shanghai Jiao Tong University, Shanghai, China

**Keywords:** *Staphylococcus aureus*, biofilm, prosthetic joint infection, antibiofilm, virtual screening

## Abstract

*Staphylococcus aureus* is one of the predominant causes of periprosthetic joint infections (PJIs). Bacterial adhesion and biofilm formation are important factors in the pathogenesis of PJIs. *S*. *aureus* biofilm formation is regulated by several factors, including *S*. *aureus* regulator A (SarA). Previous studies have found that SarA mutants have limited ability to develop biofilms. In this study, we identified a SarA-targeting antibiofilm compound, ZINC00990144, and evaluated its efficacy and toxicity. According to static biofilm assay, the antibiofilm ability of the compound was concentration dependent. ZINC00990144 reduced biofilm in multiple strains by 40–86% at a concentration of 11.5 μM. Additionally, ZINC00990144 inhibited biofilm formation on different orthopedic implant materials including Titanium and UHMWPE disc. Furthermore, quantitative polymerase chain reaction results demonstrated that ZINC00990144 upregulated the expression of *S*. *aureus* exoproteases to inhibit the formation of biofilms. Moreover, ZINC00990144 prevented biofilm formation when exposed to sub-inhibitory doses of vancomycin, which is known to promote biofilm formation. CCK-8 results demonstrated ZINC00990144 has no significant effect on cell viability at concentration of 11.5 μM or below. Finally, we verified the antibiofilm function of the compound *in vivo* using implant infection mice model with/without exposure to sub-inhibitory vancomycin. In conclusion, ZINC00990144 acts by modulating between biofilm and planktonic state of *S*. *aureus* instead of being bactericidal. Therefore, it has the potential to be used in combination with other antibiotics to prevent PJIs.

## Introduction

Biofilms are a consortium of microbial communities, such as bacteria, that are attached to each other as well as a surface and are surrounded by extracellular matrix (ECM), including proteins, exopolysaccharides, and extracellular DNA (eDNA) (Le et al., 2014; Moormeier and Bayles, 2017). In contrast to their planktonic state, bacteria in a biofilm are more recalcitrant to the host immune system and antimicrobial therapy (Figueiredo et al., 2017; Ricciardi et al., 2018). The formation of biofilms within the human body and on the surface of implanted medical devices can be extremely devastating. Additionally, as the use of implanted medical devices continues to increase, implant infection cases will also continue to rise.

Periprosthetic joint infection, a devastating complication after arthroplasty, is the main cause of arthroplasty revisions (20–36.1%) (Kapadia et al., 2016; Postler et al., 2018; Kokko et al., 2019). Patients with PJI face poor prognosis and huge economic burden. Although the microbiological epidemiology of PJIs varies from country to country, the most common pathogen responsible for PJIs is *Staphylococcus aureus* (Fernandes and Dias, 2013). Patients with *S*. *aureus* PJI have a poor prognosis (Lourtet-Hascoet et al., 2016). Given the frequent emergence of multidrug resistant strains, antibiotics alone are inadequate for PJI treatment, thus, emphasizing the need for development of antibiofilm drugs for combinatorial therapy (Ciofu et al., 2017).

Staphylococcal accessory regulator A, a global regulator, controls the transcription of a range of virulence genes by binding to the promotor region of its target genes. It has been reported that SarA mutations limit biofilm formation under both *in vivo* and *in vitro* conditions (Trotonda et al., 2005; Abdelhady et al., 2014). Inspired by this phenomenon, Balamurugan and Rekha (Arya and Princy, 2013; Arya et al., 2015; Balamurugan et al., 2017) designed 13 SarAIs as antibiofilm compounds. Both studies used *de novo* computer-assisted drug design methods based on SarA amino acid residues—DER (D88, E89, R90), a highly conserved amino acid sequence among the SarA family members (Liu et al., 2006). However, Liu et al. (2006) showed that R84 residue is also critical for DNA binding. Therefore, we propose that R84 should also be considered when carrying out *in silico* drug design.

In this study, we screened several drug-like compounds for their antibiofilm property. The compound with the best antibiofilm activity, ZINC00990144, was selected for experimentation. It is known that subinhibitory doses of antibiotics (e.g., vancomycin) can induce *S. aureus* biofilm formation. Hence, we investigated whether ZINC00990144 could inhibit *S. aureus* biofilm stimulated by sub-MIC vancomycin. We investigated the cytotoxicity of the compound via CCK-8 cytotoxicity assay. We also studied its efficacy in a mouse subcutaneous model of implant-associated infection.

## Materials and Methods

### Virtual Screening for SarA Inhibitors

The crystal structure of SarA (PDB ID: 2frh) was downloaded from the Protein Data Bank database. The conserved residues R84, D88, E99, and R90 of the SarA family were determined via multi-sequence alignment using ClustalW (Larkin et al., 2007) and the result was displayed via Jalview1.8. A compound library from Specs database^1^ containing 316,044 drug-like molecules was chosen for screening. We used Autodock Vina 1.1.2 program for structure-based virtual screening of SarAIs. The docking grid box was centered on the conserved residues to encompass all the important residues. The energy range and exhaustiveness were set at 3 and 8, respectively.

### Bacterial Strains and Compound Preparation

*Staphylococcus aureus* strains involved in this study were either maintained by our laboratory or isolated from PJI prosthesis. To construct a fluorescence labeled strain, pCM29 (Pang et al., 2010) plasmid with superfolder green fluorescent protein (sfGFP) reporter system was introduced into *S. aureus* competent cells RN4220 via electroporation and maintained using chloromycetin (10 μg/mL). Next, the plasmid was transformed into *S*. *aureus* ST1792 isolated from infectious prosthesis with bacteriophage11. Detailed strain information is listed in Table 1.

**TABLE 1:** Strains used in this study*.

Strain name	Antibiotic resistance	Description
ST1792^#^	MSSA	Isolated from PJIs patients
ST239^&^	MRSA	Isolated from PJIs patients
USA300	MRSA	CA-pneumonia
RN4220	MSSA	Restriction minus and modification plus strain
PJI_32	MRSA	Isolated from PJIs patients
PJI_27	MRSA	Isolated from PJIs patients
PJI_18	MRSA	Isolated from PJIs patients
PJI_55	MSSA	Isolated from PJIs patients
PJI_58	MSSA	Isolated from PJIs patients
PJI_57	MSSA	Isolated from PJIs patients
RN4220-sfGFP	MSSA	Fluorescence labeled strain
ST1792-sfGFP	MSSA	Fluorescence labeled strain

*All of the strains are grown in TSBG at 37°C.^#^MLST type 1792.^&^MLST type 239.

All the compounds were acquired from Specs^1^ and dissolved in dimethyl sulfoxide (DMSO) at concentration of 12.8 mg/mL for storage. During the experiment, the storage solution was diluted with culture medium (in the case of *in vitro* experiment) or normal saline (in the case of *in vivo* part) according to the dilution ratio.

### *In vitro* Static Biofilm Assays

All bacterial strains involved in this study were cultured at 37°C overnight in TSBG, and the culture was serially diluted to a concentration of ∼1 × 10^6^ colony forming units/mL (CFU/mL); the serially diluted bacterial cells (200 μL) were inoculated in a 96-well plate and the plate was incubated at 37°C for 24 h. The culture was aspirated from each well, and the wells were washed gently thrice with 200 μL of PBS to remove the non-adherent cells. After fixation with methanol, the plate was air-dried and the biofilm was stained with 200 μL of crystal violet. The biofilm biomass at the bottom of the well was dissolved in 200 μL of 33% acetic acid, and 100 μL aliquot from each well was transferred into a new 96-well plate. Optical absorbance was measured at 590 nm with a microplate reader (BioTek Instrument, Inc., United States) to quantify the biofilm biomass. For biofilm formation on Titanium disc or UHMWPE disc, bacteria were incubated with the material at 37°C overnight in TSBG.

To investigate whether protein is indispensable in biofilm matrix, proteinase K (Beyotime, China) was added to TSBG at a final concentration of 100 μg/mL to eliminate proteins as described previously (Moormeier et al., 2014). Bacteria were cultured overnight and biofilm biomass was quantified using crystal violet staining as described above.

### Confocal Laser Scanning Microscopy (CLSM)

Biofilms were cultured overnight at 37°C in TSBG as described above. The supernatant was removed gently, and the biofilm mass that remained at the bottom of the well was stained with the Live/Dead BacLight bacteria viability kit (Invitrogen, United States) according to the instructions of the manufacturer. Live (stained with green fluorescent dye Syto9) and dead (stained with red fluorescent dye propidium iodide) cells were viewed with CLSM (Leica TCS SP8, Germany).

### *S. aureus* Growth Curve

A final concentration of 11.5 μM ZINC00990144 in TSBG was incubated with *S. aureus* USA300 (∼1 × 10^6^ CFU/mL) at 37°C overnight, and the OD600 of the cells was measured every 2 h until it reached the stationary phase.

### CCK-8 Cytotoxicity Assay

HFF-1 (human foreskin fibroblasts) were pre-incubated to ∼80% convergence in DMEM containing 10% FBS and penicillin-streptomycin in a 96-well plate (∼10,000 cells/well) at 37°C, 5% CO_2_. The supernatant was then removed and 100 μL culture medium with either 23, 11.5, or 5.73 μM ZINC00990144, respectively, was added. After incubation for 24 h, the culture medium was removed and 100 μL of culture medium with 10 μL CCK-8 solution (Dojindo, Japan) was added to each well. The plate was incubated for 1.5 h in an incubator at 37°C (Thermo Fisher Heracell, United States), and the optical absorbance was measured at 450 nm with a microplate reader (BioTek Instrument, Inc., United States).

### *S. aureus* RNA Isolation and qRT-PCR

To investigate the expression of genes regulated by SarA, *S*. *aureus* was cultured overnight in TSBG with or without 11.5 μM ZINC00990144. The bacterial cells were harvested and transferred to a tissuelyser (50 Hz, 30 s) to physically disrupt the cell wall. Then the RNeasy Mini kit (Qiagen, Germany) was used to isolate RNA according to the manufacturer’s instructions. RNA with 260 nm/280 nm > 2.0 (Nanodrop, Thermo Fisher Scientific, United States) was used for reverse transcription. Fresh RNA was immediately reverse transcribed into cDNA using an RT-PCR kit (Takara, Dalian, China). cDNA was used as the DNA template for real-time PCR (Takara, Dalian, China). The primers used in this study are summarized in Table 2. Relative gene expression level was quantified using the 2^–ΔΔ*Ct*^ method (Livak and Schmittgen, 2001) with *gyrB* as the internal reference. Each group contained three independent replicates.

**TABLE 2:** qPCR Primers used in this study.

Primer name	Sequence (5′–3′)
gyrB_F	TTGGTACAGGAATCGGTGGC
gyrB_R	TCCATCCACATCGGCATCAG
aur_F	ATGGTGATGGTGATGGTCGC
aur_R	TTGACATGCTGCGTAAAGCG
sspA_F	CGCAGTCAAGCAAACAGCAA
sspA_R	CCTACAACTACACCGGAAGCA
sspB_F	ACGGTAAATCACAAGGCAGAGA
sspB_R	AGCGCATGTCCTAAATGTGG
splA_F	CCCGGAAAAGAAGACCTTGC
splA_R	TTTCACTTTTGCTCCGTCTGC
splB_F	GGCAGGGGCTAAAGCTGGTG
splB_R	TCTACTGACATCACAGGGCCAG
splc_F	TGCAGTCGTTGAAGAGACACA
splc_R	CACCGTTTGGATGGGCAGTA
splD_F	GGCAGCTCTGGTTCACCTAT
splD_R	ACCTTGTACTTTCACCTGTTGGT
splE_F	AACCAGGCAACTCAGGTTCAG
splE_R	TATTTCCAGGGCCGTTTCCAC

### Anti-biofilm Efficacy in Mice Implant Infection Model

Mice handling and related procedures in this study were reviewed and approved by the Animal Care and Ethics Committee of Sixth People’s Hospital affiliated to Shanghai Jiao Tong University, China.

The effect of inhibitors was assessed in a murine model of implant infection. Forty adult male C57BL/6 mice were randomly divided into four groups. To construct an implant infection model, a titanium disc was implanted into the dorsal area subcutaneously, and ∼1 × 10^6^ CFU of strain ST1792 were inoculated around the metal disc. Two groups were treated with DMSO (*n* = 10) or sub-MIC vancomycin (*n* = 10) and were set as control. SarAI-treated groups with (*n* = 10) or without (*n* = 10) sub-MIC vancomycin exposure were chosen as experimental groups. To observe the *in vivo* biofilm structure, we used sfGFP labeled *S*. *aureus* (RN4220-sfGFP/ST1792-sfGFP) to construct the mouse model. All treatments for this strain were performed as with strain ST1792 as described above. All the mice were euthanized 7 days after infection. Peri-implant tissues were harvested and homogenized in 1 mL sterile saline before CFU counting. The biofilm on the titanium discs was either observed under fluorescence microscope (Leica DMI8, Germany) (in the case of strain RN4220/ST1792-sfGFP) or sonicated in 1 mL sterile saline for further bacterial load quantification (in the case of strain ST1792).

### Statistical Analysis

Statistical analysis was performed using GraphPad prism 7. The data are expressed as mean ± standard deviation. Student’s *t*-test was used for normally distributed data. Bonferroni–Dunn method was used to correct for multiple comparisons. Statistical significance level was defined as a two-tailed *p* < 0.05.

## Results

### Identification of Candidate SarA Inhibitors

We aligned different SarA family proteins (UniProt accession numbers in parenthesis): SarX (Q2G0D1), SarA (Q7A1N5), SarS (Q2G1N7), SarU (Q2G1T7), SarT (Q2G2B1), SarR (Q9F0R1), and SarV (Q2FVY9) to identify the most conserved amino acids of the active site of SarA protein; L40, K82, R84, D88, and R90 were the most conserved amino acid residues (Supplementary Figure 1). According to previous reports (Liu et al., 2006), R84, D88, E89, and R90 are critical for the activity of SarA. Therefore, we selected R84, D88, E89, and R90 as the target sites for biofilm inhibition and conducted a virtual screening of the known inhibitors. Among 3,160,144 drug-like molecules, our virtual screening program revealed a set of new potential SarAIs that were different from those reported in previous studies. The candidate inhibitors were ranked according to their binding affinity to the target sites. Finally, we purchased top 23 compounds (Specs^2^) for the biological assays (Supplementary Table 1).

### *In vitro* Evaluation of Potential SarA Inhibitors

In our preliminary trial (Supplementary Figure 2), all the compounds were used at a concentration of 1.28 mg/mL. Microtiter plate biofilm assay showed that most of the compounds could inhibit *S*. *aureus* biofilm formation (approximately 15.47–55.9%), while ZINC00990144 exhibited significant (*p* < 0.001) ability to inhibit biofilm formation (approximately 68.3%). Since ZINC00990144 had outstanding biofilm inhibition ability, it was chosen for further experiments.

**FIGURE 1:**
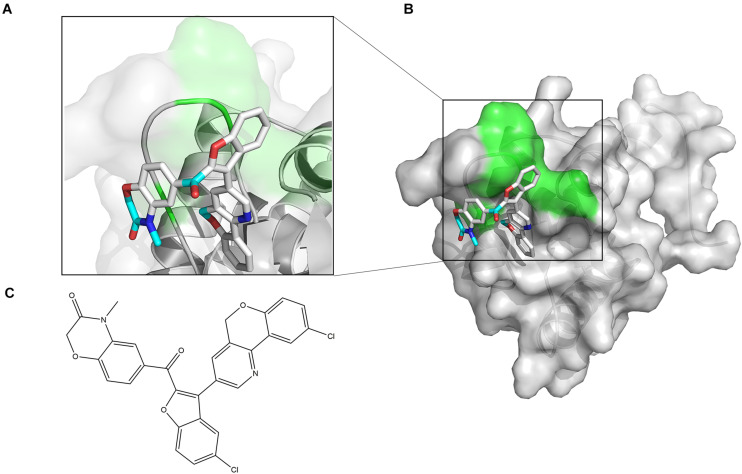
Computational model of compound ZINC00990144 bound to SarA revealed by docking simulations. **(A)** SarA proteins are shown in cartoon mode with conservative residues marked with green and labeled. ZINC00990144 is displayed as stick. Light blue: carbon, dark blue: nitrogen, gray: hydrogen, and red: oxygen. **(B)** Protein surfaces are shown in gray and predicted binding sites are marked with green. **(C)** Structure of ZINC00990144.

The chemical structure of ZINC00990144 was shown in Figure 1C. Predicted binding mode with SarA revealed that ZINC00990144 blocked the active site residues of SarA protein (Figures 1A,B).

### Exploring the Best Concentration for Usage

**FIGURE 2:**
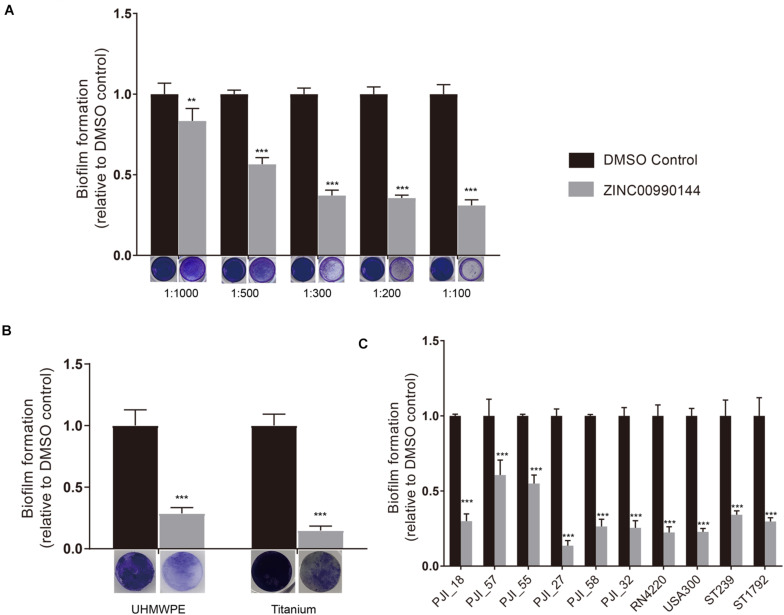
Concentration gradient of ZINC00990144 on the inhibition biofilm formation **(A)**. Biofilm inhibition potency of ZINC00990144 on different materials **(B)** and against different *S. aureus* strains **(C)**. **(A)** The significance of biofilm inhibition increased with the increment of concentration in strain USA300. Biofilm was stained with crystal violet and quantified by measuring 590 nm absorbance. Dilution ratio is relative to storage solution. (For example: 1:1000 represents 2.3 μM compound or 0.1% DMSO). **(B)** USA300 biofilm formation on UHMWPE and titanium are significantly prevented by ZINC00990144 administration at the concentration of 11.5 μM (dilution ratio 1:200). Representative gross image of UHMWPE and titanium implant are shown under the *x*-axis. **(C)** ZINC00990144 at concentration of 11.5 μM is effective to all of the *S. aureus* isolates we tested. Biofilm was quantified with crystal violet staining method. **P < 0.01, ***P < 0.001 comparing to DMSO control.

We diluted the compound serially and repeated the microtiter plate biofilm assay as described above. The result showed that the antibiofilm ability was concentration dependent (Figure 2A). Briefly, concentration as low as 2.3 μM (1:1000 dilution) was enough to produce observable effects and a concentration of 11.5 μM (1:200 dilution) was close to its maximum function. As a result, the inhibitor was used at a concentration of 11.5 μM (dilution ratio 1:200) for further experiments.

### Anti-biofilm Function on Different Materials and Different *S. aureus* Strains

In artificial joint implants, the most used materials are titanium alloy and UHMPWE. Compared to untreated materials, biofilms on both materials could be efficiently inhibited by 11.5 μM concentration of ZINC00990144 (Figure 2B, *p* < 0.001).

Furthermore, we selected eight *S*. *aureus* strains isolated from PJIs prosthesis and two most used *S*. *aureus* strains (strains USA300 and RN4220) for analysis. Although the sensitivity to ZINC00990144 varied among the strains, they were still susceptible to ZINC00990144 (Figure 2C). In addition, both MRSA and MSSA strains were susceptible to ZINC00990144.

### Observation of the Structure of Biofilms via CLSM

**FIGURE 3:**
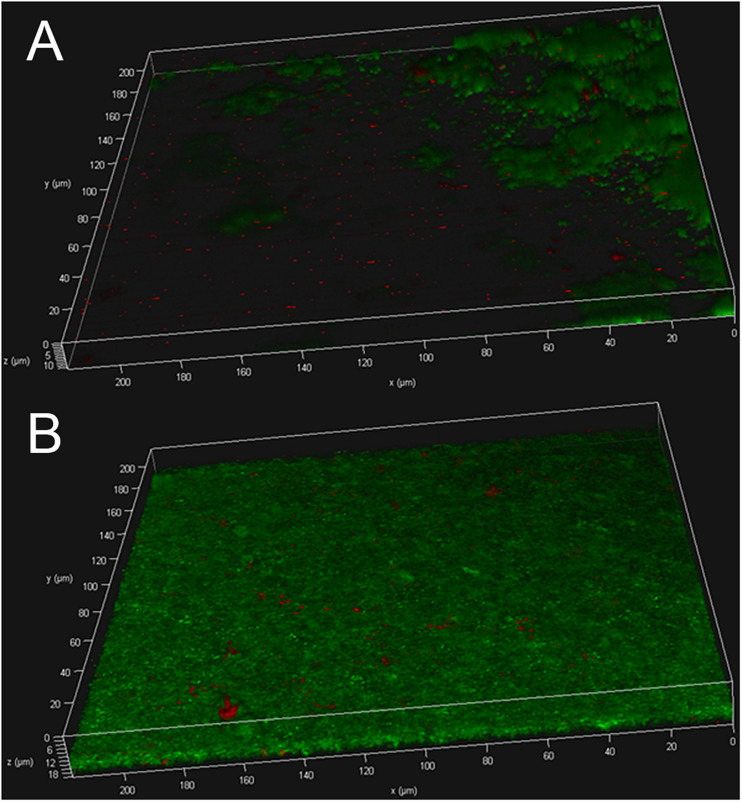
USA300 biofilm with **(A)** or without **(B)** ZINC00990144 treatment was observed under CLSM after live/dead staining.

The biofilms were further subjected to live/dead staining. As shown in Figures 3A,B strain USA300 forms a robust biofilm with a thickness of up to ∼12 μm after 12 h of incubation in TSBG, most of the cells are alive with propidium iodide-labeled dead cells (red) scattered among the biofilm. On the other hand, treatment with ZINC00990144 significantly affected the biofilm forming ability of strain USA300. Mature biofilm was not observed in most of the observation fields, while small clusters of bacteria were occasionally detected. The thickness of the bacterial clusters displayed high variation, ranging from ∼3 to ∼12 μm.

### qPCR Analysis for Genes Regulated by SarA

**FIGURE 4:**
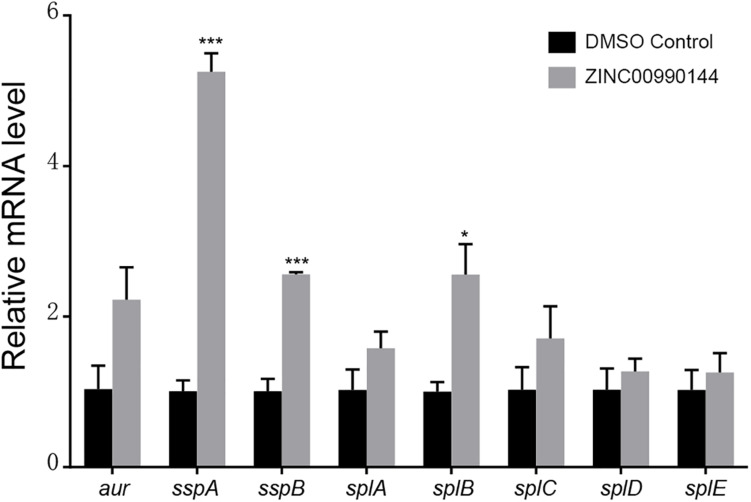
Transcriptional changes of selected biofilm-associated genes after ZINC00990144 treatment. Following ZINC00990144 treatment, transcription levels of *aur*, *sspA*, *sspB*, *splA*, *splB*, *splC*, *splD*, and *splE* were investigated by qPCR in strain USA300. Each group contains three independent replicates. The fold change was in relative to the untreated samples. *P < 0.05, ***P < 0.001 comparing with DMSO control.

Agnieszka et al. (Zielinska et al., 2012) reported that SarA mediated proteases are closely correlated with decreased biofilm formation. We used qPCR to explore whether ZINC00990144 influenced the transcription of the reported extracellular proteases (*aur*, *sspA*, *sspB*, *splA*, *splB*, *splC*, *splD*, *splE*). In comparison to the control, a 5.25-fold, 2.56-fold, and 2.56-fold increase in *sspA*, *sspB*, and *splB* transcription, respectively, was observed due to SarAI treatment (Figure 4).

### SarAI Prevents Sub-MIC Vancomycin Induced Biofilm Formation

**FIGURE 5:**
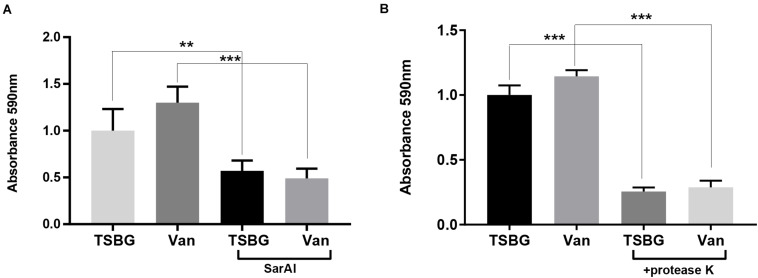
Anti-biofilm function of ZINC00990144 **(A)** and proteinase K **(B)**. **(A)** ZINC00990144 is effective to USA300 biofilm with/without exposure to sub-MIC vancomycin. **(B)** Sub-MIC vancomycin promotes biofilm formation. Proteinase K (100 μg/mL) added at the initiation of the experiment inhibited biofilm formation completely, regardless of whether vancomycin is present. Van: sub-MIC vancomycin (0.5 μg/mL), SarAI: ZINC00990144, TSBG: TSB medium supplied with 0.5% glucose, **P < 0.01, ***P < 0.001 comparing with corresponding control.

When exposed to sub-MIC vancomycin, *S. aureus* biofilm formation was slightly promoted compared with the controls without exposure to vancomycin (Figures 5A,B), though the difference was not remarkable. In addition, *S*. *aureus* subjected to SarAI (with/without vancomycin) exhibited a significant reduction in biofilm formation (Figure 5A) (*p* < 0.01/*p* < 0.001 for groups with/without vancomycin, respectively).

### Protein Is Essential in *S. aureus* Biofilm Development

In order to investigate the role of protein in biofilm development, Proteinase K was added in TSBG to eliminate proteins. The result showed that addition of exogenous proteases exhibited significant biofilm clearance with/without exposure to sub-MIC vancomycin (*p* < 0.001) (Figure 5B).

### Cytotoxicity and Bactericidal Effects of ZINC00990144

The cytotoxicity of ZINC00990144 was evaluated at concentrations ranging from 6.75 to 23 μM (dilution ratio ranging from 1:400 to 1:100, Figure 6A). ZINC00990144 at concentrations of 6.75 and 11.5 μM had no observable effects on cell growth. However, concentration of 23 μM (relative cell viability 89 ± 1.5%) and its corresponding DMSO control (1%) exhibited cell toxicity (relative cell viability 75.7 ± 4.8%) when comparing to blank control.

**FIGURE 6:**
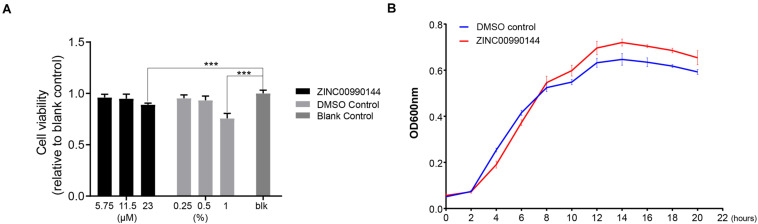
Cytotoxicity of ZINC00990144 to HFF-1 cell line **(A)** and *S. aureus* **(B)**. **(A)** No significant difference detected between untreated (blank control) and treated group at 5.75 and 11.5 μM concentrations of ZINC00990144 by CCK-8 assay. 23 μM ZINC00990144 exhibits slight cytotoxicity which could be attributed to the solvent when comparing with the DMSO control. 5.75,11.5, 23 μM ZINC00990144 contains 0.25, 0.5, 1% DMSO, respectively. **(B)** ZINC00990144 does not hamper the growth of USA300. ***P < 0.001 comparing to blank group.

By analyzing the growth curve of *S. aureus*, ZINC00990144 treated group did not inhibit the growth of *S*. *aureus* (Figure 6B). Instead, it slightly promoted *S*. *aureus* proliferation during the stationary phase.

### Role of ZINC00990144 in Mice Model of Implant Infection

**FIGURE 7:**
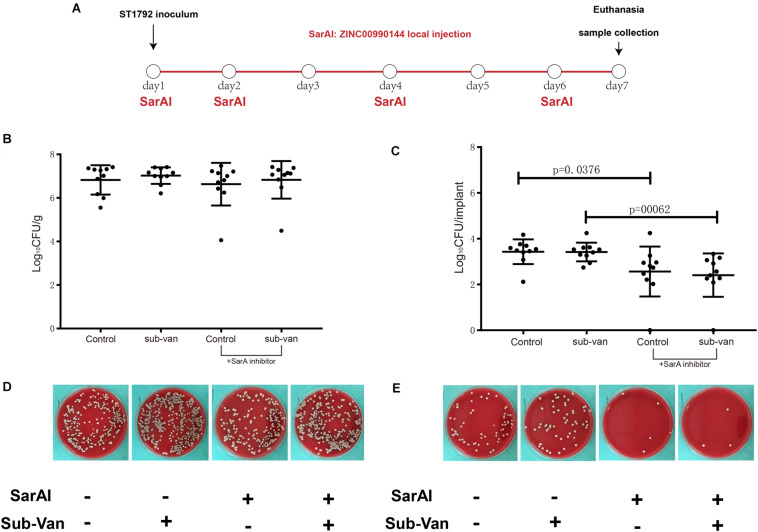
Role of ZINC00990144 in the mice model of implant infection. **(A)** Schematic diagram for process of *in vivo* experiment. SarA inhibitor was used in implant infection mice model at day1, 2, 4, 6. All of the mice were sacrificed at day 7. **(B–E)** Quantification of bacterial load for the implant and surrounding infected tissue. **(B,D)** Bacteria count on surrounding tissues. **(C,E)** Bacteria count on implant and representative photos.

In a mouse model of implant infection, intervention was given at specific time points as described in Figure 7A. All mice (*n* = 40) were euthanatized 7 days after infection. When mice were not exposed to sub-MIC vancomycin, SarAI treated group showed a 0.864 log CFU reduction (*p* < 0.05) in adherent bacterial count compared with control group. Similarly, when sub-MIC vancomycin was presented, SarAI also reduced adherent bacteria by 1.011 log CFU (*P* < 0.001) compared to the control (Figures 7C,E). However, the peri-implant bacterial load had no significant difference among the four groups (Figures 7B,D).

**FIGURE 8:**
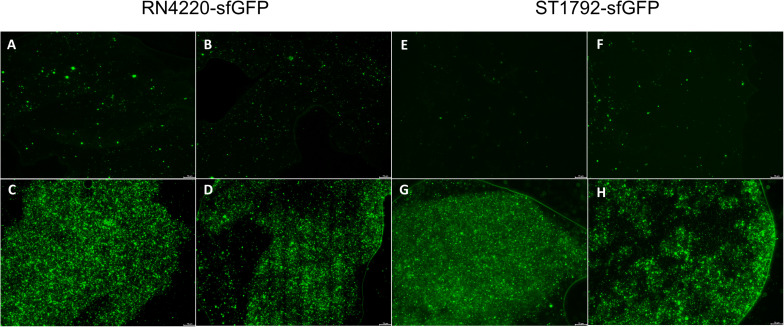
Representative fluorescence images of biofilm *in vivo*. Titanium discs was harvested on seventh day since infection and observed under microscope. Green fluorescence represents sfGFP labeled RN4220 **(A–D)** and ST1792 **(E–H)**. **(A,E)** Mice model given SarA inhibitor ZINC00990144. **(B,F)** Mice model given ZINC00990144 and sub-MIC vancomycin. **(C,G)** Control mice model without sub-MIC vancomycin. **(D,H)** Mice model given sub-MIC vancomycin. Scale bar = 75 μm.

We also inoculated the implant infection mice model with sfGFP labeled strains and the titanium discs were collected for observation (Figure 8). Green fluorescence was sparsely dispersed in the treatment group (SarAI with or without sub-MIC concentration of vancomycin). Some of the bacteria adhered to the surface of the titanium disc in single cell state, but some aggregated together to form bacterial clusters. In contrast, the groups without SarAI intervention formed typical mature biofilms surrounded by a clear border; in these groups, green fluorescence labeled *S*. *aureus* cells were densely distributed and existed in aggregation.

## Discussion

In this study, we adopted virtual screening method and screened a list of potential antibiofilm molecules by targeting *S. aureus* regulator SarA. It has been widely reported that SarA mutants have attenuated biofilm formation. Previously, Arya and Princy (2013) discovered 13 potential SarAIs by employing computer-assisted *de novo* drug design method with the software LUDI (Bohm, 1992). Two of the potential inhibitors were evaluated *in vitro* or *in vivo*, and both showed remarkable antibiofilm effect (Arya et al., 2015; Balamurugan et al., 2017). Different from *de novo* drug design, which designs new molecules from scratch, virtual screening identifies potential ligands from existing chemical compound databases. In this study, the compound library was obtained from Specs database, and all compounds complied with the Lipinski’s rule of drug-likeness (also known as rule of five, Ro5) (Lipinski et al., 2001).

According to the predicted binding mode between ZINC00990144 and SarA protein (Figures 1A,B), ZINC00990144 blocked the promoter binding region of SarA protein. However, there is no molecular force such as hydrogen bond or van der waals force observed between SarA and ZINC00990144. Further optimization of ZINC00990144 to increase binding force may improve its potency. SarA down-stream gene transcription analysis shows that SarA negatively regulated protease was upregulated in ZINC00990144 treated group (Figure 4). It also supports ZINC00990144 as an SarAI.

In Orthopedics, vancomycin is one of the commonly used antibiotics in bone cement for infection treatment and prevention. However, antibiotics in cement are always explosively released and the concentration rapidly reduces over time. When the concentration of vancomycin drops under the MIC concentration, it promotes *S. aureus* biofilm formation (Hsu et al., 2011; Abdelhady et al., 2014), our results also verified this phenomenon (Figures 5A,B). It has been reported Staphylococcus biofilm could be divided into PIA-dependent and PIA-independent biofilm (proteinaceous biofilm) (Rohde et al., 2007). Previous studies have demonstrated that vancomycin promotes biofilm formation by inducing PIA production (Hsu et al., 2011; Abdelhady et al., 2014). Since ZINC00990144 prevent biofilm formation by upregulating protease and degrading protein, we speculated proteinaceous biofilm would be more susceptible to ZINC00990144 than PIA-dependent biofilm such as sub-MIC vancomycin induced biofilm. However, our study found that ZINC00990144 efficiently inhibited sub-MIC induced biofilm which demonstrated ZINC00990144 is also potent toward PIA-dependent biofilm (Figure 5A). Previously studies (Mootz et al., 2013; Moormeier et al., 2014) revealed that protein is essential for *S. aureus* biofilm integrity and adhesion/multiplication process. Taken these findings into consideration, the underlying mechanism could be explained by the hypothesis that protein is equally important in PIA-dependent and PIA-independent biofilm. According to our result, exogenous proteases efficiently inhibited sub-MIC vancomycin induced biofilm (Figure 5B). This result combined with previous reports demonstrated protein is an indispensable part both in PIA-dependent/independent biofilm. Strategies targeting proteins could be an effective way to limit biofilm formation.

In our study, ZINC00990144 presented anti-biofilm ability in a concentration-dependent mode and concentration at 11.5 μM is close to its maximum function (Figure 2A). Comparing with previous reported SarAIs, of which the effective concentration is 2.5 μM (Balamurugan et al., 2017), ZINC00990144 is still not an ideal inhibitor and needs optimization. Moreover, our results show that ZINC00990144 is highly effective toward different *S. aureus* strains including both MRSA and MSSA (Figure 2C), it is also functional on different materials such as titanium and UHMWPE (Figure 2B), which are widely used in arthroplasty, all of this together endows ZINC00990144 promising applications in a wide range of fields.

As for cytocompatibility, ZINC00990144 had a slight cytotoxicity effect when concentration reaches 23 μM (Figure 6A). However, it is still unclear whether the toxicity was from compound itself or the solvent (DMSO), because the corresponding DMSO control group (1%) also exhibits cytotoxicity. In this study, most of the experiment was carried out at concentration of 11.5 μM which has no cytotoxicity according to our result; hence, ZINC00990144 has good cytocompatibility at its effective concentration.

Despite the effective potency of the compound toward biofilm inhibition, our study had some limitations. First, the most suitable dose for treatment was around 11.5 μM; such a high concentration cannot be easily orally administered, therefore, we adopted local injection when conducting *in vivo* experiments. However, local injection cannot maintain the concentration at an effective level for a long duration because molecules are rapidly absorbed and metabolized by the body. Thus, selecting a drug delivery system for slow-release is necessary, such as nanoemulsions, lipid or polymeric nanoparticles, and liposomes (Shi et al., 2010). Second, the compound we identified cannot eliminate already existing mature biofilms, indicating its role in prophylaxis rather than therapy. Moreover, in consideration of its non-bactericidal effect, it should be regarded as an adjuvant compound for combinatorial usage. Besides, the *in vivo* experiment in our study only investigated early stage biofilm at a single time point and the implant infection model was soft tissue related instead of bone related. Thus, it may not truly reflect the disease process of PJI.

Although we found ZINC00990144 has an impact on SarA-associated phenotypes, it is not a direct demonstration that ZINC00990144 inhibits SarA function because many other *S. aureus* regulatory loci are known to impact these phenotypes. In order to directly investigate the function of SarA, several other assays could be adopted in the future. First, since SarA functions by binding to DNA regions, electrophoretic mobility shift assay (EMSA) is the most effective assay investigating SarA function. Second, a Luciferase reporter system governed by a known SarA binding promoter is also an alternative approach to reflect SarA activity. These methods are commonly adopted by researchers to evaluate the transcription factor activity.

In summary, here we identified a new SarA targeted anti-biofilm leading compound using virtual screening method and testified its potency *in vivo* using implant infection mice model. As an anti-biofilm compound without bactericidal effects, ZINC00990144 can be used in combination with other bactericidal antibiotics.

## Data Availability Statement

The raw data supporting the conclusions of this article will be made available by the authors, without undue reservation.

## Ethics Statement

The animal study was reviewed and approved by the Animal Care and Experiment Committee of Shanghai Jiao Tong University Affiliated Sixth People’s Hospital.

## Author Contributions

JLY contributed to the concept of the study, performed virtual screening, and wrote the manuscript. FJ and FYZ contributed to the qPCR experiment and cell culture. YQP performed the biofilm assay. JT and JQW collected clinical strains and performed antibiotic susceptibility testing. JLY, FJ, and FYZ performed the *in vivo* experiment and data acquisition. PH contributed to data analysis and data interpretation. HS contributed to study design and manuscript editing and revision. All authors contributed to the article and approved the submitted version.

## Conflict of Interest

The authors declare that the research was conducted in the absence of any commercial or financial relationships that could be construed as a potential conflict of interest.

## Footnotes

1 www.specs.net

2 www.specs.net

## Supplementary Material

Supplementary Figure 1 Multiple alignment result for SarA family. Seven SarA family members are aligned using clustalW. The color depth represents conservative amino acid residues.

Supplementary Figure 2 *In vitro* static biofilm assays for all purchased 23 compounds. All of the compounds except ZINC00969968 could inhibit biofilm with varied extent. Among all of the compounds, compound ZINC00990144 shows excellent anti-biofilm performance. ^∗∗∗^*P* < 0.001 comparing to DMSO control.

## Abbreviations

MIC: minimum inhibitory concentration
MLST: multilocus sequence typing
PIA: polysaccharide intercellular adhesion
PJI: periprosthetic joint infection
SarA: Staphylococcal accessory regulator A
SarAI: SarA inhibitor
TSBG: tryptic soy broth supplied with 0.5% glucose
UHMWP: ultra high molecular weight polyethylene.
