# Supplementary figures and images for: Virtual Screening for Novel SarA Inhibitors to Prevent Biofilm Formation of Staphylococcus aureus in Prosthetic Joint Infections
Source: Front Microbiol. 2020 Nov 5;11:587175. doi: 10.3389/fmicb.2020.587175 (PMC7674313; doi:10.3389/fmicb.2020.587175)

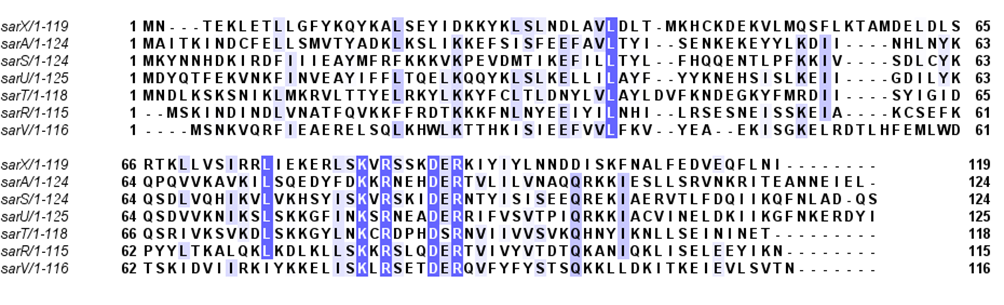

Supplement: Supplementary Figure 1 — Multiple alignment result for SarA family. Seven SarA family members are aligned using clustalW. The color depth represents conservative amino acid residues. [file Image_1.TIF]
